# Enhancer RNAs step forward: new insights into enhancer function

**DOI:** 10.1242/dev.200398

**Published:** 2022-08-30

**Authors:** Laura J. Harrison, Daniel Bose

**Affiliations:** Molecular and Cellular Biology, School of Biosciences, Sheffield Institute For Nucleic Acids, The University of Sheffield, Firth Court, Western Bank, Sheffield S10 2TN, UK

**Keywords:** eRNA, Non-coding RNA, Enhancers, Chromatin, Transcription, Gene expression

## Abstract

Enhancers confer precise spatiotemporal patterns of gene expression in response to developmental and environmental stimuli. Over the last decade, the transcription of enhancer RNAs (eRNAs) – nascent RNAs transcribed from active enhancers – has emerged as a key factor regulating enhancer activity. eRNAs are relatively short-lived RNA species that are transcribed at very high rates but also quickly degraded. Nevertheless, eRNAs are deeply intertwined within enhancer regulatory networks and are implicated in a number of transcriptional control mechanisms. Enhancers show changes in function and sequence over evolutionary time, raising questions about the relationship between enhancer sequences and eRNA function. Moreover, the vast majority of single nucleotide polymorphisms associated with human complex diseases map to the non-coding genome, with causal disease variants enriched within enhancers. In this Primer, we survey the diverse roles played by eRNAs in enhancer-dependent gene expression, evaluating different models for eRNA function. We also explore questions surrounding the genetic conservation of enhancers and how this relates to eRNA function and dysfunction.

## Abstract (precis)

**Summary:** This Primer evaluates the ideas that underpin developing models for eRNA function, exploring cases in which perturbed eRNA function contributes to disease.

## Introduction

The complex architecture of mammalian chromosomes directs spatial interactions between transcriptional elements to establish gene regulatory networks (Sexton et al., 2009; Tolhuis et al., 2002). Enhancers underpin these gene regulatory networks, conferring precise spatiotemporal patterns of gene expression in response to developmental and environmental stimuli. As such, their activity is highly specific to distinct cell lineages and stimuli. Enhancer activity involves the interplay between enhancers (*cis*-regulatory elements) and *trans*-acting factors, such as transcription factors (TFs), in the context of the local chromatin environment. Simplistically, enhancer potential is determined by the binding of TFs to DNA and by the packaging of DNA into chromatin (Miller and Widom, 2003). Indeed, enhancers are characterised by the co-occurrence of sequence-specific TF binding sites and DNA sequences associated with nucleosome depletion (Khoueiry et al., 2010; Spitz and Furlong, 2012; de Almeida et al., 2022). However, enhancer potential also relies on a myriad of factors that control chromatin accessibility, for example DNA methylation and histone post-translational modifications (PTMs) (Calo and Wysocka, 2013).

Over the last decade, the transcription of enhancer RNAs (eRNAs) – nascent RNA species pervasively transcribed from active enhancers – has emerged as a key factor that contributes to enhancer activity (De Santa et al., 2010; Kaikkonen et al., 2013; Kim et al., 2010; Hah et al., 2011; Li et al., 2013). The transcriptional landscape at enhancers is complex, generating eRNAs with diverse structures, directionality and stability. Due to this heterogeneity, the categorisation of enhancer-derived transcripts poses a challenge, and the definitions of eRNAs and characteristically similar long non-coding RNAs (lncRNAs) are blurred (Box 1). Furthermore, some regulatory lncRNAs function in a transcript-independent manner (Engreitz et al., 2016), whereas others function via the act of transcription itself (reviewed by Kaikkonen and Adelman, 2018). eRNAs tend to be relatively short-lived, transcribed at very high rates but also quickly degraded, such that their half-lives are often of the order of minutes (Rabani et al., 2014; Schwalb et al., 2016). Nevertheless, eRNA production is deeply intertwined within enhancer regulatory networks and is thus implicated in the transcriptional control of gene expression.

**Box 1. Characteristics of eRNAs**

The majority of identified ‘classical’ eRNAs are short (∼200nt-2 kb), bidirectionally transcribed transcripts that are 5′ m^7^G capped, non-polyadenylated and non-spliced (Andersson et al., 2014; De Santa et al., 2010; Kim et al., 2010; Kristjánsdóttir et al., 2020; Schwalb et al., 2016). Longer (up to 4-5 kb), polyadenylated and spliced, unidirectionally transcribed eRNAs – often referred to as enhancer-associated lncRNAs – have also been identified (De Santa et al., 2010; Djebali et al., 2012; Gil and Ulitsky, 2018; Koch et al., 2011; Vučićević et al., 2015; Hah et al., 2013). For shorter transcripts, eRNA length is dictated by the Integrator complex, which processes and controls the length of a number of short RNA species (Lai et al., 2015; Lykke-Andersen et al., 2021). It is unclear whether the differences between classical eRNAs and enhancer-associated lncRNAs confer any distinct functions, but distinct transcripts are likely to display differences in transcript stability (Gil and Ulitsky, 2018). The sensitivity of RNA transcripts to degradation by the RNA exosome decreases as the distance between the transcription start site (TSS) and the polyadenylation site increases. Thus, the production of short eRNAs may prevent the complete assembly of the polyadenylation machinery on RNAPII, resulting in suboptimal polyadenylation and subsequently RNA exosome intervention (Ntini et al., 2013). This may explain the propensity for longer eRNAs to be polyadenylated while the majority of short eRNAs are non-polyadenylated and unstable, subject to rapid degradation. The transience of short, non-polyadenylated eRNA sequences likely limits their spatial range of action, whereas the absence of such strict temporal restriction may allow longer, polyadenylated eRNAs to survey the nuclear space in search of target sequences, potentially in *trans* (De Santa et al., 2010; Kim and Shiekhattar, 2016; Tsai et al., 2018).

Although gene expression patterns and TF binding preferences are largely conserved across mammals (King and Wilson, 1975; Nitta et al., 2015), enhancers show changes in function and sequence over evolutionary time. Such a dichotomy raises questions about the relationship between enhancer sequence and enhancer function, especially when considering eRNAs. For example, how do you reconcile a functionally important eRNA with a lowly conserved sequence? In addition, the vast majority of single nucleotide polymorphisms (SNPs) associated with human complex diseases, and many of those associated with cancer, map to the non-coding genome, with causal disease variants enriched within *cis*-regulatory elements, particularly enhancers (van Arensbergen et al., 2019; Bal et al., 2022; ENCODE Project Consortium, 2012; Farh et al., 2015; Hindorff et al., 2009). Such localisation of disease variants within enhancers supports the importance of sequence for regulating enhancer function. However, it is also known that enhancers exhibit promiscuous behaviours – a single enhancer can govern different effects in different cell types (Fukaya et al., 2016) – suggesting that enhancer function is unlikely to be driven by intrinsic sequence features alone.

In recent years, we have begun to establish an understanding of the roles of eRNAs. However, the lines separating the independent functions of enhancers from the functions of eRNAs are becoming increasingly blurred. This Primer surveys the diverse roles played by eRNAs in enhancer-dependent gene expression, evaluating different models for eRNA function and exploring the questions they pose in relation to genetic conservation at enhancer elements and the role of enhancer sequences in disease.

## The effects of eRNAs on the chromatin environment

Gene expression patterns are determined by the actions of enhancers in the context of the native chromatin environment. In eukaryotes, histone modifications and DNA methylation confer epigenetic control over chromatin environments and, therefore, gene expression. Together, they alter chromatin density and thereby accessibility of DNA to the basal transcriptional machinery, modulating the transcriptional potential of regulatory DNA elements (Cedar and Bergman, 2009). Concordantly, the deposition of characteristic histone modifications on flanking nucleosomes, and DNA hypomethylation, are universal features of active enhancer elements (Heintzman et al., 2009; Lister et al., 2009; Rada-Iglesias et al., 2011; Stadler et al., 2011). eRNAs likewise can contribute to gene expression by regulating access of transcriptional machinery at defined genomic loci. Upon eRNA depletion, a reduction in chromatin accessibility at promoters is observed, suggesting that eRNAs may facilitate nucleosome rearrangement and/or recruitment of chromatin remodellers (Mousavi et al., 2013).

### eRNAs activate histone acetylation

Enhancers harbour characteristic patterns of histone modifications, so distinctive in fact that the location of putative enhancers can be identified genome-wide by mapping histone modifications enriched at enhancers. Acetylation of lysine 27 on histone H3 (H3K27ac) and monomethylation of lysine 4 on histone H3 (H3K4me1) are the predominant histone modifications deposited at the nucleosomes flanking enhancer elements. H3K4me1 is globally associated with enhancers (Heintzman et al., 2007), independent of activity, whereas the presence of H3K27ac is characteristic of active enhancer elements (Creyghton et al., 2010; Rada-Iglesias et al., 2011). It is no surprise that enhancers, as major sites of combinatorial TF assembly, are also occupied by transcriptional co-activators such as BRD4, and by the histone acetyltransferases CREB-binding protein (CBP) and p300. These are responsible for enhancer-associated histone modifications such as H3K27ac and have been used for the genome-wide annotation of enhancer elements (Heintzman et al., 2009; Kim et al., 2010; Rada-Iglesias et al., 2011; Visel et al., 2009). Deposition of histone acetylation by p300/CBP and other acetyltransferases bestows dynamic control over enhancer activation and stimulates the expression of target genes (Hilton et al., 2015; Miao et al., 2022). Notably, p300/CBP inhibition abrogates enhancer activation, coupled with the rapid attenuation of eRNA expression levels, highlighting p300/CBP-dependent acetylation as a key driver in enhancer activation (Narita et al., 2021). Additional modifications of histones are also associated with enhancers, particularly those that are implicated in nucleosome dynamics and chromatin opening to promote transcription, such as H3K64ac, H3K122ac and H4K16ac (Di Cerbo et al., 2014; Pradeepa et al., 2016; Simon et al., 2011; Taylor et al., 2013; Tropberger et al., 2013), broadly implicating chromatin opening and transcription as a key feature of active enhancers. CBP directly interacts with eRNAs at active enhancers, an interaction that stimulates the acetyltransferase activity of CBP and results in RNA-dependent modulations of H3K27ac (Bose et al., 2017; Carullo et al., 2020). These interactions underlie changes in CBP activity, modulating histone acetylation and thus fine-tuning target gene expression. Notably, limited sequence conservation is observed in the eRNAs that bind to CBP; specificity instead arises from the binding of specific, local populations of eRNAs to CBP (Bose et al., 2017). These results suggest that the precise sequence of an eRNA may be less important than the presence of the eRNA transcript itself and hint at a general mechanism for eRNA function.

### The effects of eRNAs on repressive histone methylation

Although H3K27ac is generally considered an activating histone modification, tri-methylation of the same residue (H3K27me3) is a crucial repressive modification during development, ensuring genes that require silencing are packaged into heterochromatin. H3K27me3 is generated by polycomb repressive complex 2 (PRC2) (Ferrari et al., 2014). In embryonic stem cells (ESCs), PRC2 binds at repressed developmental regulator genes (Tanay et al., 2007) and promiscuously interacts with nascent RNAs at essentially all active genes (Beltran et al., 2016; Wang et al., 2017a). RNA binding effectively antagonises the histone methyltransferase activity of PRC2, alleviating chromatin of repressive histone modifications and activating gene expression (Cifuentes-Rojas et al., 2014; Kaneko et al., 2013). eRNAs may share this function: knockdown of eRNAs transcribed from the distal enhancer of the gonadotropin hormone ɑ-subunit (*Cga*) gene converts H3K27 from an active acetylated state to a repressive methylated state at both the enhancer and the associated proximal promoter (Pnueli et al., 2015). Moreover, the eRNA *CARMN*, which plays a crucial role in cardiovascular cell fate determination and differentiation (Ounzain et al., 2015), physically interacts with PRC2 to mediate epigenetic regulation of the cardiac commitment programme (Ounzain et al., 2015), akin to other cardiogenic lncRNAs, such as braveheart (*Bvht*) and *Fendrr* (Grote et al., 2013; Klattenhoff et al., 2013).

PRC2 preferentially binds RNA motifs composed of short repeats of consecutive guanine residues; the natural abundance of this RNA motif explains the promiscuous binding activity of PRC2. Further, PRC2-DNA binding sites are significantly enriched for sequences that code for PRC2-binding RNA motifs, potentially mediating site-specific RNA interactions. In line with its preference for guanine tracts, PRC2 binds to guanine quadruplex (G-quadruplex) structures in RNA with high affinity (Wang et al., 2017b). This posits a model in which secondary structures in eRNAs promote active transcription from enhancers, firstly by inhibiting DNA methylation and secondly by sequestering PRC2 from nucleosome substrates, leading to H3K27me3 depletion and subsequently gene activation (Beltran et al., 2019; Mao et al., 2018). Thus, DNA demethylation and PRC2 removal may be encoded within the DNA sequence to promote gene activation at associated loci (Wang et al., 2017a,b).

**Fig. 1.**
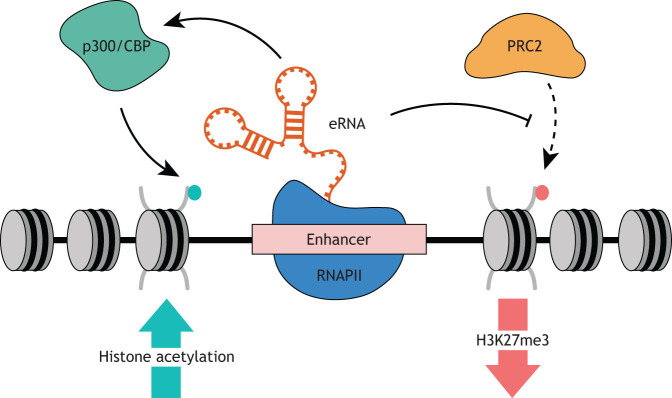
**eRNA interactions with chromatin modifying enzymes at enhancers.** Enhancer RNAs (eRNAs) are RNA species that are pervasively transcribed from active enhancers. They interact with histone modifying enzyme complexes at enhancers resulting in altered patterns of histone modifications at these sites. For example, eRNA interactions with PRC2 and p300/CBP inhibit the deposition of repressive H3K27me3 PTMs and promote the deposition of activating H3K27ac PTMs, respectively, helping to maintain an open chromatin state at enhancers and promoting further eRNA synthesis.

Together, these studies of H3K27ac and H3K27me3 highlight the potential of eRNAs to interact with enzyme complexes responsible for generating histone modifications – both activating and repressive – to generate a chromatin environment that is conducive to active transcription (Fig. 1). At active enhancers, although eRNAs may bind to PRC2 to inhibit its histone methyltransferase activity (Beltran et al., 2016; Cifuentes-Rojas et al., 2014), they may also stimulate the acetyltransferase activity of CBP (Bose et al., 2017). This suggests that eRNAs promote a feed-forward loop, whereby enhancer activation yields eRNA production that, in turn, maintains open chromatin at the enhancer to reinforce enhancer activation. Moreover, without nascent eRNA transcripts antagonising PRC2 activity, H3K27me3 and thus repressive chromatin is successfully maintained at inactive enhancers (Beltran et al., 2016; Cifuentes-Rojas et al., 2014).

### eRNAs and DNA methylation

DNA methylation acts as a dynamic gene regulatory mechanism, encoding both transient and permanent changes in gene regulatory activity. Cytosine methylation, typically at CpG dinucleotides, is generally repressive (Ehrlich and Wang, 1981), whereas hypomethylated CpG regions – CpG islands – favour transcription (Deaton and Bird, 2011). Enhancers display greater variation in their pattern of methylation between different tissues, cell types and developmental states than promoters (Bogdanović et al., 2016; Hon et al., 2013; Ziller et al., 2013). eRNA production correlates with DNA hypomethylation on a genome-wide basis and DNA hypomethylation is characteristic of active enhancers (Pulakanti et al., 2013). Hypomethylated, actively transcribed enhancers are occupied by TET hydroxylases, which actively demethylate CpG at enhancers to promote a transcriptionally active environment (Pulakanti et al., 2013; Stadler et al., 2011). For example, TET2-mediated demethylation occurs pervasively throughout reprogramming of induced pluripotent stem cells (iPSCs) and, at enhancers, precedes the formation of open chromatin (Sardina et al., 2018). Moreover, short hairpin RNA (shRNA)-mediated depletion of TET1 or TET2 reduces eRNA levels to 30-60% of endogenous levels at *Nanog*-linked enhancers in ESCs (Pulakanti et al., 2013).

In hepatocellular carcinoma (HCC), the methylation status of the CCAAT/enhancer-binding protein-β (*CEBPB*) enhancer directly impacts tumorigenicity. Hypomethylation of the *CEBPB* enhancer induces eRNA synthesis and is correlated with poor prognosis in HCC patients (Xiong et al., 2019). *CEBPB* eRNAs exhibit pro-tumorigenic properties via methylation-regulated control of *CEBPB* transcription, and their depletion significantly reduces tumour cell growth and invasiveness. Concordantly, *CEBPB* eRNA levels are significantly elevated in HCC tumours compared with non-tumour tissues (Xiong et al., 2019).

## eRNAs as direct transcriptional regulators

Although the above examples illustrate the role of eRNAs in indirectly regulating transcription by modulating the chromatin environment, more direct roles for eRNAs as regulators of transcription have also been demonstrated.

### Effects on RNA polymerase II pause release

RNA polymerase II (RNAPII) pausing is a genome-wide regulatory mechanism by which the pausing and subsequent release of RNAPII into productive elongation acts as a key rate-limiting step for transcription. eRNAs can facilitate the release of paused RNAPII, thus promoting the transition into productive elongation, via interactions with complexes of negative elongation factor (NELF) and the positive elongation factor P-TEFb (Schaukowitch et al., 2014; Shii et al., 2017). P-TEFb is a multiprotein complex composed of cyclin-dependent kinase 9 (CDK9) and cyclin T1. eRNAs interact with P-TEFb by directly binding to cyclin T1; the resultant activation of P-TEFb promotes the phosphorylation of NELF, DRB sensitivity inducing factor (DSIF) and Serine 2 of the RNAPII C-terminal domain (CTD), via the pause-releasing kinase activity of CDK9 (Fujinaga et al., 2004; Shii et al., 2017; Wada et al., 1998; Yamaguchi et al., 1999; Zhao et al., 2016). P-TEFb-catalysed phosphorylation sites on the NELF-A subunit of the NELF complex overlap with eRNA binding sites identified on this subunit (Gorbovytska et al., 2022). Phosphorylation of such sites is essential for RNAPII pause release (Lu et al., 2016) and is hypothesised to induce a conformational change in NELF conducive to NELF release from the paused elongation complex. In line with this, eRNA binding within the vicinity of P-TEFb target sites may trigger a similar conformational change that bypasses P-TEFb activity altogether. Consequently, NELF phosphorylation by P-TEFb and eRNA binding may similarly bestow a negative charge to NELF that facilitates a conformational change and, ultimately, results in dissociation of NELF from the paused elongation complex (Gorbovytska et al., 2022).

Such interactions between eRNAs and NELF have been shown to be crucial for rapid induction of neuronal immediate early genes (IEGs) in neurons. In this context, eRNA binding to NELF facilitates the release of paused RNAPII in response to neuronal stimulation (Gorbovytska et al., 2022; Schaukowitch et al., 2014). Replacing wild-type NELF-E with an RNA recognition motif (RRM) deletion mutant in neurons causes a reduction in NELF binding at IEG promoters and mRNA induction (Schaukowitch et al., 2014). In this case, eRNAs bind directly to a canonical RNA binding motif in the NELF-E subunit. However, NELF-E binds to a variety of RNA sequences with little or no sequence or structural constraint (Yamaguchi et al., 2002), suggesting that mutations in the eRNAs would have minimal effect on the efficiency of eRNA binding to NELF. This is another example of the eRNA transcript itself rather than a particular RNA sequence being the driver of eRNA function.

### eRNAs and transcription factor binding

As well as regulating transcription initiation directly, eRNAs can interact with a variety of transcriptional activators and repressors. For example, eRNAs can bind to the multi-functional TF yin yang 1 (YY1), which functions as both a transcriptional activator and a repressor (Shi et al., 1997). eRNAs bind to YY1, increasing its occupancy at enhancers, via the zinc fingers of YY1 and with no strong RNA sequence preference (Sigova et al., 2015; Wai et al., 2016). Nascent RNA transcribed from both enhancers and promoters may help to capture dissociating YY1, increasing its occupancy (Sigova et al., 2015). This ‘TF trapping’ activity may be a common mechanism of action for eRNAs, as eRNAs also increase the binding of other TFs such as c-Jun and NF-κB at their target loci (Huang et al., 2018; Shii et al., 2017; Spurlock et al., 2017). Transcriptional control may therefore involve an additional positive-feedback loop in which YY1 and other TFs stimulate local enhancer transcription, and newly transcribed eRNAs reinforce local TF occupancy, contributing stability to gene expression *in vivo* (Sigova et al., 2015). YY1 can also recruit PRC2 to specific loci, promoting the deposition of repressive H3K27me3 (Atchison et al., 2003; Caretti et al., 2004; Pan et al., 2013; Wilkinson et al., 2006). However, RNA interactions with PRC2 via the EZH2 subunit effectively inhibit its methyltransferase activity (Cifuentes-Rojas et al., 2014). Thus, eRNAs may both promote YY1, and consequently PRC2, occupancy but may also downregulate the activity of recruited PRC2, providing another example of eRNAs fine-tuning transcriptional outputs.

eRNAs can also bind to the transcriptional and epigenetic regulator BRD4, which plays a well-established role in cancer development (Donati et al., 2018). In human colorectal cancer, BRD4 is recruited to enhancers that are linked to proinflammatory gene expression patterns and co-occupied by mutant p53 upon chronic TNF-ɑ signalling (Rahnamoun et al., 2017; 2018). Together, BRD4 and p53 cooperate to support synthesis of eRNAs and expression of tumour-promoting genes (Rahnamoun et al., 2018). eRNAs synthesised from these enhancers bind to BRD4 via its tandem bromodomains, enhancing and stabilising BRD4 binding to acetylated histones and active enhancers. Here, eRNAs support interactions between BRD4 and acetylated histones to maintain enhancer and gene activation (Rahnamoun et al., 2018). BRD4 demonstrates largely promiscuous RNA binding activity but, similar to CBP, binds eRNAs in a locus-specific manner such that it selectively associates with eRNAs produced from specific BRD4-bound enhancers (Rahnamoun et al., 2018; Bose et al., 2017; Carullo et al., 2020).

## The role of eRNA structure in regulating eRNA function

Although multiple studies have demonstrated functional roles for eRNAs in transcriptional regulation, the role played by specific eRNA sequences and conserved eRNA structures is less well defined. Following the classification of ∼10,000 newly detected nascent RNAs, including 3257 classified as eRNAs, ∼10% were predicted to be structured (Schwalb et al., 2016). However, the roles played by these RNA structures is relatively poorly understood. Below we highlight examples in which the function of an eRNA has been linked to its structure.

The ^DRR^eRNA (also known as MyoD upstream non-coding RNA or MUNC) is transcribed from the enhancer regions of the myogenic master regulator MyoD (Cichewicz et al., 2018; Tsai et al., 2018). ^DRR^eRNA directs cohesin loading in *trans* to promote chromatin opening during myogenesis, implicating ^DRR^eRNA as a regulator of muscle cell differentiation. ^DRR^eRNA is recruited to the *Myog* locus in *trans*, where it colocalizes with nascent transcripts, positing the formation of RNA:RNA interactions between the ^DRR^eRNA and *Myog* nascent transcripts as a potential in *trans* target recognition mechanism (Tsai et al., 2018). Chemical probing studies enabled modelling of ^DRR^eRNA secondary structure, revealing the presence of multiple well-defined functional domains, with different domains mediating distinct features of ^DRR^eRNA promyogenic activity (Przanowska et al., 2022). Notably, ^DRR^eRNA does not regulate the expression of all of its target genes by a common mechanism; instead, distinct combinations of effector domains are required for the induction of different target genes, thus demonstrating that the functional complexity of ^DRR^eRNA is integrated within its complex secondary structure (Przanowska et al., 2022).

As mentioned above, eRNAs can regulate RNAPII pause release by binding to NELF. They exert their dissociative function on NELF in a manner that is more dependent on eRNA length than overt structure. To trigger the dissociation of NELF from the paused elongation complex, a single eRNA containing unpaired guanosines (but not G-quads, in contrast to PRC2) forms multiple simultaneous allosteric contacts with several RNA binding sites on NELF. In the absence of sequence and structure dependency, the length of the eRNA, and therefore the degree of eRNA flexibility, is key, facilitating folding of the eRNA substrate into a structure that favours simultaneous binding to both the NELF-E and NELF-A subunits (Gorbovytska et al., 2022).

However, although eRNA binding to NELF-E (Rao et al., 2006; Yamaguchi et al., 2002) does not require structure specificity, RNA structure is important for eRNA binding to P-TEFb, where eRNA-mediated activation of P-TEFb promotes target gene transcription via phosphorylation of Serine 2 of the RNAPII CTD. (Richter et al., 2002; Zhao et al., 2016). Here, a TAR RNA-like (TAR-L) motif in the androgen receptor-regulated eRNA (*AR*-eRNA) shares a similar secondary structure with the 3′ end of the small nuclear RNA *7SK* (also known as *RN7SK*) (Zhao et al., 2016), an established inhibitor of P-TEFb (Nguyen et al., 2001; Yang et al., 2001). Competitive binding of the *AR*-eRNA displaces *7SK* and activates P-TEFb (Zhao et al., 2016). As *AR*-eRNA and *7SK* exhibit a common secondary structure, the interactions between them and P-TEFb are probably due to structural recognition rather than sequence specificity.

**Fig. 2.**
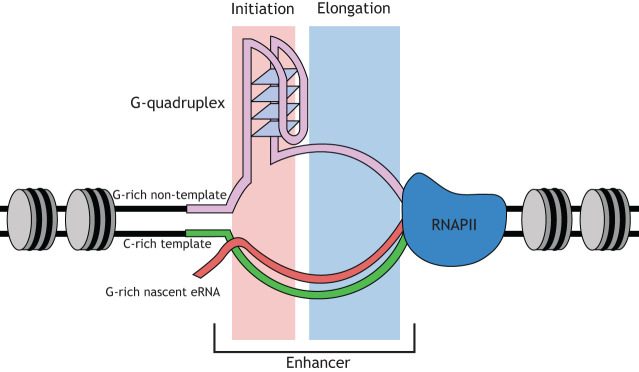
**R-loops and DNA G-quadruplexes at enhancers.** DNA/RNA hybrid structures, termed R-loops, are regions in which a nascent RNA anneals to the template strand of the DNA duplex. R-loop formation occurs preferentially at consecutive clusters of guanine residues in the non-template DNA strand within the R-loop initiation zone. Downstream of this, high-density G-rich sequences in the R-loop elongation zone facilitate extension of the RNA:DNA hybrid. R-loop formation is also dependent on the stability of the resulting RNA:DNA hybrid and the unhybridised single-stranded (ss)DNA stretch. G-richness in the non-template strand imparts greater stability to R-loop structures; in particular, clusters of G tracts can fold into stable G-quadruplex structures that can stabilise the displaced DNA strand.

eRNAs can also co-transcriptionally assemble into DNA/RNA hybrid structures, termed R-loops (Fig. 2), in which the nascent RNA anneals to the template strand of the DNA duplex (Pefanis et al., 2015; Wulfridge and Sarma, 2021; Yan et al., 2019). R-loop formation is associated with enhancer-like epigenetic signatures, including an open chromatin state, the deposition of characteristic histone modifications and unmethylated DNA (Ginno et al., 2012; 2013; Kuznetsov et al., 2018; Nadel et al., 2015; Sanz et al., 2016). R-loop formation is determined by nucleic acid sequence, with G-rich RNA hybridising with C-rich DNA in the template strand (Ratmeyer et al., 1994), and is favoured by the presence of G-quadruplexes on the non-template strand of the DNA duplex (Kuznetsov et al., 2018; Lim and Hohng, 2020; Wulfridge and Sarma, 2021; Ginno et al., 2012, 2013; Li and Manley, 2005; Ratmeyer et al., 1994). G-quadruplexes are also enriched within enhancer DNA (Hou et al., 2021 preprint) and it is thought that G-quadruplexes help to stabilise the R-loops formed by eRNAs and contribute to maintaining the nucleosome-depleted regions necessary for sustained enhancer activity (Hou et al., 2021 preprint; Maizels and Gray, 2013; Wulfridge and Sarma, 2021).

## Linking eRNA structure to disease

RNA secondary structure is sensitive to mutation (Halvorsen et al., 2010). As such, changes in eRNA structure may link disease-associated SNPs and genetic variation at enhancers to altered molecular function and phenotypic effects. Key to linking RNA structure to phenotype is the ability to predict single nucleotide variants (SNVs) that generate large RNA structural disparities, commonly termed riboSNitches (Halvorsen et al., 2010; Wan et al., 2014). Genome-wide prediction of RNA structure approaches have identified a multitude of known and novel RNA structures within eRNA regions, with stem-loops being the most common motif (Ren et al., 2017). The novel predicted structures demonstrate similarity to transfer RNAs (tRNAs) and the stem-loop structure of the microRNA miR-155: such structural similarities may imply similar molecular functions (Ren et al., 2017). Further, inflammatory autoimmune disease-associated genetic variants are disproportionately associated with riboSNitches in eRNA regions, implying a mechanism whereby disease-associated SNPs contribute to the aetiology of autoimmune diseases through RNA structure (Ren et al., 2017). In prostate cancer cells, for example, the cytosine-rich domain of antisense *AR*-eRNA, which is predicted to form a stem-loop structure, is key for *AR*-eRNA function, recruiting DNA methyltransferase 1 (DNMT1) to the associated enhancer to alter the DNA methylation levels of *AR* target loci (Pan et al., 2021).

In estrogen receptor α (ERα)-positive breast cancer cells, genome-wide annotation of 17β-estradiol (E2)-regulated eRNAs identified a ∼40 nucleotide functional motif predicted to form a hairpin loop structure, termed the FERM element. The FERM element in eRNAs transcribed from ERα-bound enhancers modulates the expression of the cognate target genes, *PRRX2* and *UBE2E2*. The eRNAs work in a locus-specific manner to promote recruitment of ERα and stimulate p300-catalysed H3K27ac. Importantly, mutations within the FERM element of the *PRRX2* eRNA that abolish secondary structure ablate the stimulatory effect of the eRNA on ERα binding, H3K27ac and target gene expression (Hou and Kraus, 2022). The FERM element also interacts with breast carcinoma-amplified sequence 2 (BCAS2) (Hou and Kraus, 2022), a coregulator of ERα-mediated transcription implicated in breast cancer (Qi et al., 2005; Salmerón-Hernández et al., 2019). BCAS2 knockdown abolishes the stimulatory effect of the FERM motif eRNAs, consequently altering target gene expression. *PRRX2* eRNA and mRNA expression levels are correlated, and targeting the *PRRX2* eRNA to its cognate enhancer promotes breast cancer cell proliferation, an effect that is abrogated upon depletion of BCAS2. Overall, this suggests that eRNAs can act at oncogenic enhancers to enhance oncogenic gene expression, via stimulation of co-regulator activities, to ultimately promote cancer cell proliferation (Hou and Kraus, 2022).

Roles for eRNAs in enhancer-promoter looping have also been observed at the locus encoding oncogenic heparinase (HPSE), a protein that is upregulated in various malignancies and bestows metastatic potential to cancer cells (Hulett et al., 1999; Shinyo et al., 2003). *HPSE* eRNAs interact with the RNA-binding protein heterogeneous nuclear ribonucleoprotein U (hnRNPU) via its arginine/glycine-rich RGG domain – a canonical RNA binding motif. This binding induces structural alterations in hnRNPU that favour its interactions with p300; the subsequent enrichment of hnRNPU and p300 at the enhancer promotes looping to the HPSE promoter and stimulates *HPSE* expression (Jiao et al., 2018). It should be noted that RGG domains are intrinsically disordered, which facilitates promiscuous binding to a myriad of RNA species (Järvelin et al., 2016). However, RGG domains are not entirely indiscriminate and demonstrate moderate preference for GC-rich sequences and RNAs with complex secondary structures, including G-quadruplexes. Therefore, the promiscuous RNA binding activity of RGG domains is likely a consequence of preference for RNA sequences and structures that are exhibited frequently throughout the transcriptome (Ozdilek et al., 2017).

## Transcriptional condensates and eRNAs

Phase-separated transcriptional condensates have been shown to form at enhancers, generating high local concentrations of transcriptional regulators and promoting their synergistic function (Hnisz et al., 2017; Shrinivas et al., 2019). The disordered domains that promote condensate formation are often sites of sequence-independent interactions with RNA and form the basis of a prevalent category of non-canonical RNA binding motifs (Castello et al., 2016; He et al., 2016; Hentze et al., 2018). Unsurprisingly, therefore, transcriptional condensates are enriched in and dependent on RNA, which scaffolds and modulates their morphology and composition (Banani et al., 2016). Many properties of RNA might promote their involvement in the formation of phase-separated biomolecular condensates (Box 2). This compartmentalisation of RNAs and proteins into sub-organellar condensates may compensate for the lack of sequence specificity in RNA-protein interactions. Notably, high intrinsic disorder in proteins is a prerequisite for condensate formation and many factors that bind to eRNAs do so through disordered regions (Boija et al., 2018; Cho et al., 2018; Sabari et al., 2018; Roden and Gladfelter, 2021).

**Box 2. RNA structure and condensate formation**

Assembly of the transcriptional machinery into biomolecular condensates at enhancers is a tightly-regulated process that is dependent on both RNA sequence features, such as RNA identity, length and modifications, and RNA properties intrinsically encoded in their sequence, such as RNA structure (Roden and Gladfelter, 2021). For example, the negative charge of RNA promotes complex coacervation – the demixing of oppositely-charged macromolecular species such as polymers and proteins (Drobot et al., 2018; Frankel et al., 2016; Pak et al., 2016). Such dependence on electrostatic interactions may explain the largely sequence-independent actions of diverse RNAs in phase-separated condensates (Drobot et al., 2018; Frankel et al., 2016). However, phase separation characteristics may not be entirely independent of sequence. Poly(A), poly(C) and poly(U) RNAs are unlikely to form persistent tertiary structures and tend to form dynamic, liquid-like condensates when interacting with disordered protein models. In contrast, poly(G)-rich RNA sequences form highly stable G-quadruplexes (Petrovic and Polavarapu, 2008) and have a higher propensity to form a fractal network of structures characteristic of kinetically arrested gel-like condensates (Boeynaems et al., 2019; Sciortino et al., 1993). Condensate formation may also be influenced by additional, partially sequence-encoded RNA features, such as RNA modifications and RNA:RNA interactions (Langdon et al., 2018; Ries et al., 2019).

Multiple examples exist of condensate-associated diseases caused by alterations in primary RNA sequence, the best-studied being the hexanucleotide repeat expansion within the *C9orf72* gene in familial Amyotrophic Lateral Sclerosis (ALS) and Frontotemporal Dementia (FTD) (DeJesus-Hernandez et al., 2011; Renton et al., 2011). In this example, the disease-associated GGGGCC (G_4_C_2_) repeat expansion templates multivalent interactions, driving the formation of large gel-like aberrant foci via phase separation (Jain and Vale, 2017). Interestingly, it has been observed that the disease-associated G_4_C_2_ repeat found in the *C9orf72* locus can also readily form G-quadruplexes (Conlon et al., 2016; Reddy et al., 2013). Such stable G-quadruplex structures sequester the splicing regulator protein, hnRNPH, into insoluble aggregates within the brain, resulting in splicing defects in hnRNPH-regulated transcripts, contributing to neurodegeneration (Conlon et al., 2016).

A model is emerging in which the transition from transcriptional initiation into elongation is driven by the shuttling of RNAPII between distinct transient condensates at gene promoters and within gene bodies, whereby condensates function to concentrate factors required for initiation and elongation, respectively (Cramer, 2019; Guo et al., 2019). Such self-compartmentalisation of transcription occurs in a phosphoform-specific manner dependent on regulatory CDKs (Guo et al., 2019). Phosphorylation of the RNAPII CTD promotes the exchange of RNAPII from condensates involved in transcription initiation, containing the Mediator co-activator complex (Kornberg, 2005), to those involved in RNA processing and splicing (Guo et al., 2019). Considering the established role for RNAs in phase separation, eRNAs could potentially play a functional role in phosphorylation-mediated transcriptional condensate partitioning. eRNAs interact with the Mediator complex to regulate its chromatin localisation, connecting transcriptional activators bound at enhancers with promoter-bound RNAPII (Lai et al., 2013; Taatjes, 2010; Wang et al., 2011). Although no direct interactions of eRNAs with Mediator subunits have been observed, siRNA-mediated knockdown of eRNAs results in decreased enhancer-promoter interactions and decreased occupancy of Mediator and RNAPII at target promoters (Hsieh et al., 2014; Lai et al., 2013). Conversely, direct activating interactions have been identified between eRNAs and the cyclin T1 component of P-TEFb (Zhao et al., 2016). Hyperphosphorylated RNAPII CTD can be incorporated into phase-separated condensates formed by the intrinsic disorder of P-TEFb. Cyclin T1 promotes phase separation, compartmentalising the kinase with the substrate CTD for robust hyperphosphorylation and subsequent transcriptional elongation and RNA processing (Lu et al., 2018). Overall, overlapping functions of eRNAs and condensates thus suggest roles for eRNAs in the partitioning behaviour of both initiation and elongation condensates.

**Fig. 3.**
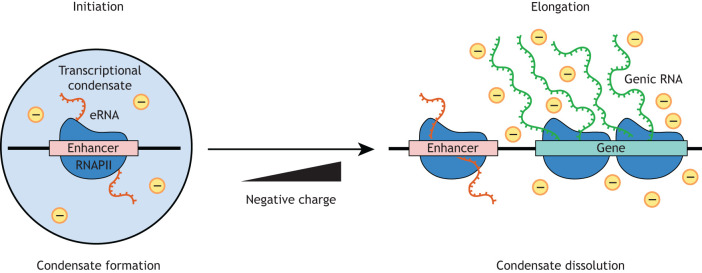
**Model of eRNA-mediated control of transcriptional condensate formation.** Early in transcription initiation, nascent eRNAs transcribed from enhancers stimulate the formation of transcriptional condensates via phase separation. Upon transition into transcriptional elongation, the production of long genic RNAs results in electrostatic repulsion that stimulates dissolution of the transcriptional condensates.

Although RNAs play a broad role in determining the formation of membraneless compartments, it has been suggested that they may be less significant for transcriptional condensate formation (Decker et al., 2022). Super-enhancers foster the crowding of TFs and transcriptional coactivators at densities conducive to transcriptional condensate formation (Shrinivas et al., 2019). However, nuclear RNA degradation has little effect on super-enhancer condensates, suggesting that eRNAs are not required for the integrity or maintenance of transcriptional condensates (Decker et al., 2022). Although eRNAs may not play a structural role in the assembly of transcriptional condensates, they may have a regulatory function in their formation and dissolution (Decker et al., 2022; Henninger et al., 2021). Early in transcription, condensate formation is driven by favourable electrostatic interactions arising from the low levels of non-coding RNAs, including eRNAs, synthesised from enhancers and promoter-proximal regions by RNAPII (Fig. 3) (Adelman and Lis, 2012; Core and Adelman, 2019; Henninger et al., 2021; Kim et al., 2010; Seila et al., 2008). RNAs may scaffold multivalent interactions between condensate components implicated in different stages of transcription, coupling distinct steps in transcription for synergistic activation (Ma et al., 2021; Narita et al., 2021), alongside directly influencing condensate dynamics to control transcriptional regulation (Henninger et al., 2021). Upon release of RNAPII into productive elongation, the synthesis of long genic RNAs leads to repulsive-like electrostatic effects that result in condensate dissolution (Banerjee et al., 2017; Henninger et al., 2021; Milin and Deniz, 2018), suggesting that short-lived eRNAs are more likely to contribute to the early stages of condensate formation than their later dissolution (Fig. 3).

## Post transcriptional modification of eRNAs

The deposition of chemical modifications on eRNAs, such as N6-methyladenosine (m6A), may affect transcriptional condensate assembly. Both the METTL3/METTL14/WTAP m6A methyltransferase complex (MTC) and the demethylase ALKBH5 associate with enhancers (Lee et al., 2021; Xu et al., 2022). Consequently, m6A is pervasively deposited on nascent eRNAs (Lee et al., 2021), and eRNA abundance is dynamically regulated by m6A methylation (Lee et al., 2021; Xu et al., 2022). Notably, m6A signals on eRNAs are highly correlated with features of active enhancers (Lee et al., 2021), suggesting that they act to promote gene expression.

Mechanistically, m6A-modified eRNAs recruit nuclear m6A reader proteins such as hnRNPG (RBMX) and YTHDC1 to active enhancers, promoting productive enhancer transcription. The protective functions of m6A readers shield eRNAs from processing by the endonuclease subunit of the Integrator complex, INTS11, which would otherwise cleave nascent RNAs, resulting in premature transcriptional termination (Xu et al., 2022). In addition, eRNA transcripts, together with YTHDC1, assemble into nuclear phase-separated condensates that can augment BRD4 coactivator condensates to modulate downstream gene expression (Lee et al., 2021).

m6A modification levels are largely hard-wired in nascent RNA sequences via the presence of ‘GGACT’ motifs, consistent with the canonical RRACH methylation motifs seen in mRNAs (Dominissini et al., 2012; Lee et al., 2021; Meyer et al., 2012). With the deposition of m6A, and potentially other chemical modifications, being dependent on short degenerate sequence motifs, such motifs may act as fundamental elements in nascent RNAs for the recruitment of reader proteins to chromatin, bestowing some level of sequence-encoded control over the transcription process.

## Perspectives

In contrast to the longstanding belief that sequence conservation implies functionality, many enhancers appear to maintain functions that are relatively independent of precise sequence conservation. This complex balance not only complicates their identification, but also raises questions regarding the mapping of disease-associated variants within enhancer elements. If enhancer sequence has little relevance to functionality, why do SNPs associated with human complex disease so frequently localise to enhancers (ENCODE Project Consortium, 2012; Farh et al., 2015; Hindorff et al., 2009)? To answer these questions, we have focussed here on eRNAs, which may act as sequence-independent determinants of enhancer activity that can promote specific transcriptional control mechanisms. For eRNAs, as for many RNAs, lack of sequence conservation does not mean a lack of functional conservation. In fact, the relative lack of sequence constraint on eRNA function appears to bestow eRNAs with the functional plasticity necessary to impact a myriad of transcriptional control mechanisms. In disease contexts, mutant enhancers potentially encode aberrant eRNAs that may disrupt this normal eRNA-mediated transcriptional regulation.

One barrier to better understanding eRNA function is the difficulty of manipulating eRNAs in the cellular environment; most studies have focused on a handful of eRNAs in isolation to characterise their function. Therefore, further studies are clearly required to examine the function of eRNAs in their native contexts. Although there is a lack of an overall conserved sequence motif for functional eRNAs, the diverse mechanisms covered in this Primer reveal a myriad of avenues by which eRNAs can exert their function in a more enhancer-specific manner. Therefore, determining whether eRNA sequence motifs and structures are fundamental in governing the functional complexity of eRNAs should be considered the focal point to unlock the roles of enhancers in gene control and disease.
